# Lectin-type oxidized LDL receptor-1 as a potential therapeutic target for cerebral cavernous malformations treatment

**DOI:** 10.3389/fnins.2024.1442110

**Published:** 2024-08-21

**Authors:** Karthik Ashok, Tyra Martinez, Julie Sesen, Sana Nasim, Shih-Shan Lang, Gregory Heuer, Alexander Tucker, Miguel Alejandro Lopez-Ramirez, Edward R. Smith, Aram Ghalali

**Affiliations:** ^1^Vascular Biology Program, Boston Children’s Hospital, Boston, MA, United States; ^2^Department of Surgery, Harvard Medical School, Boston, MA, United States; ^3^Division of Neurosurgery, Children’s Hospital of Philadelphia, Philadelphia, PA, United States; ^4^Department of Neurosurgery, Perelman School of Medicine, University of Pennsylvania, Philadelphia, PA, United States; ^5^Department of Medicine, University of California, San Diego, CA, United States; ^6^Department of Neurosurgery, Boston Children’s Hospital, Boston, MA, United States

**Keywords:** cerebral cavernous malformations, CCM, lectin-type oxidized ldl receptor 1 (LOX-1), LOX-1, urinary biomarker, proximity extension assay (PEA), Olink

## Abstract

**Introduction:**

Cerebral cavernous malformations (CCMs) are pathologic lesions comprised of clusters of thin-walled capillaries characterized by abnormal proliferation, angiogenesis, and bleeding secondary to somatic or germline mutations in endothelial cells. CCMs can cause headaches, seizures and/or neurological defects. There is a clinical need to develop better tools to detect CCMs and follow their progression in conjunction with the current use of neuroimaging techniques. Here we present data supporting the utility of LOX-1 (lectin-type oxidized LDL receptor 1), a 50 kDa transmembrane protein implicated in endothelial cell dysfunction and ischemia, as a putative biomarker for CCM.

**Methods:**

CCM urine samples (*n* = 23) were collected from pediatric CCM patients. Matched healthy controls (*n =* 24) were collected from pediatric patients with either Chiari I malformation or fatty filum terminale, and otherwise normal findings. All samples were collected with patient/family consent and institutional review board approval.

Samples were analyzed with Olink Proteomic Proximity Extension Assay (PEA). Differences in expression for 2,925 unique proteins were quantified between healthy control urine samples and CCM urine samples. The results were normalized, validated, and analyzed for demographic bias. In addition to urine samples, CCM tissue from patients was harvested and used to create primary cell lines for *in vitro* analysis of LOX-1 expression, in addition to immunofluorescence of lesional tissue excised at surgery.

**Results:**

ANOVA analysis of the CCM urine samples showed a statistically significant increase in LOX-1 compared to the control samples, with CCM patients exhibiting a > 5-fold increase in urinary expression. Corroborating these elevated levels of circulating marker, analysis of source tissue from surgically resected CCMs revealed that LOX-1 is increased in both CCM patient cavernoma primary cell lines and operative specimens.

**Conclusion:**

LOX-1 is involved with pathways implicated in CCM pathogenesis and our data here reveals that LOX-1 expression is significantly elevated in CCM patients as compared to matched healthy control individuals, including both source tissue from surgically excised CCMs and in analysis of samples collected from outside of the central nervous system, particularly urine. This proof-of-principle data suggests that LOX-1 may have potential utility as a target for CCM treatment and supports further investigation related to its potential mechanistic impact on CCM pathogenesis.

## Introduction

Cerebral cavernous malformations (CCMs), also known as cavernomas or cavernous angiomas, are pathologic clusters of thin, weak-walled capillaries in the central nervous system that may resemble a mulberry in appearance and can range in size from millimeters to several centimeters in diameter (Smith and Scott, 2010). Comprised largely of endothelial cells, these lesions are characterized by diminished, aberrant smooth muscle and pericytes, with compromised intracellular junctions between endothelial cells (Tanriover et al., 2013). The lesions can be solitary, typically from somatic mutations, or multiple, more commonly found in patients with germline mutations. While several different genes have been reported to be associated with the development of CCM, the majority of mutations exist within a common pathway that regulates cell–cell adhesion and motility (Kahle et al., 2023; Smith, 2024).

CCMs are important because they are common, present in as many as 1 in every 200 individuals, and with bleeding and growth over time, they can cause headache, seizure, and other neurologic deficits, including death (Otten, 1989). CCMs may become symptomatic at any time throughout life (Detwiler et al., 1997). While screening with genetic testing is possible for some familial cases, the majority (~80%) of CCM are sporadic and are only discovered after symptoms develop (Tournier-Lasserve, 2020). While novel therapies are in development, current treatment options are largely comprised of either surgical resection (and, rarely, radiation) or careful observation with serial imaging studies (such as magnetic resonance imaging, MRI; Dammann et al., 2022; Kuroedov et al., 2023). For those CCMs that are removed or radiated, the risk of recurrence remains—so in nearly all cases, long-term follow-up with imaging is often needed (Prolo et al., 2020; Bianconi et al., 2022).

MRIs are costly, typically require travel to an imaging center and—for children in particular—may require the risk of sedation or anesthesia to tolerate a scan. These limitations mean that there is a pressing clinical need to develop diagnostic and prognostic tools that may be complementary to current practice methods to improve the ability to detect and follow CCMs in both treated and observed patients. The use of non-invasive biomarkers, such as measuring the levels of specific molecules in urinary and serum samples, is cheap, safe, and able to be performed in nearly any location, reducing cost, risk, and travel for patients (Duggins-Warf et al., 2023). Recent advances in the application of non-invasive biomarkers in CNS disease has prompted our study of this approach in CCM (Carlyle et al., 2022; Bao et al., 2023).

Here we report an initial experience with the use of LOX-1 [lectin-like oxidized low-density lipoprotein (LDL) receptor-1], a transmembrane receptor found on endothelial cells which is activated in response to oxidative stress, abnormal hemodynamic flow, and inflammation—all conditions present in CCM (Chen et al., 2002; Zeya et al., 2016; Mentrup et al., 2020). Given the precedent of LOX-1 activation and increased expression in other vascular pathologies sharing common features with CCM, we hypothesized that LOX-1 levels would be increased in the setting of CCM—and that these elevated LOX-1 levels could be detected in CCM tissue, cell lines and urine, with potential utility target for CCM treatment (Pirillo et al., 2013; Kattoor et al., 2019).

## Materials and methods

### Patient population

A total of 23 CCM patients and 24 age and sex-matched control individuals were eligible for this study, with all specimens collected with institutional review board (IRB) approval. All patients were under 19 years of age at time of specimen collection and all CCM patients had a diagnosis of CCM confirmed by board certified neuroradiologist review (as part of routine clinical practice) and all tissue was confirmed as CCM by neuropathology (as part of routine clinical practice). The control population was selected based on prior publication precedent for CNS biomarker studies, with congenital findings of a Chiari I malformation or fatty filum terminale, as these patients had all undergone comprehensive imaging of their CNS, thereby confirming that they had normal brain and spine findings (ensuring that there were no occult tumors, vascular lesions, etc. that might potentially confound biomarker analysis).

### Urine collection

Pediatric patients had urine collected prior to surgery, during the same day of the procedure. Collected urine samples were immediately placed on ice for transport to the laboratory and stored in −80°C freezer, as previously described by us (Pricola Fehnel et al., 2016).

### Tissue collection

Cavernoma tissue was collected, according to IRB-approved protocols and patient consent, at time of surgical removal. Tissue samples were processed in laboratory for cell culture and/or fixed in formalin and embedded in paraffin for histology. Specimens were sourced from the Division of Neuropathology within Boston Children’s Hospital, as previously described by us (Akino et al., 2014; Pricola Fehnel et al., 2016; Sesen et al., 2024).

### OLINK

Urine samples were submitted to Firalis in Huningue, France for Olink Proteomic proximity extension assay (PEA; Carlyle et al., 2022; Li et al., 2024). Using the Exploratory 3,072-Plex Panel, the differences in expression for 2,926 unique proteins were quantified. The Olink PEA probe is designed to leverage the specificity of antibodies with the amplification-capability of DNA by using specific antibody-epitope recognition with quantitative polymerase chain reaction. Peptide-specific antibodies are bound to single-stranded oligonucleotides. Two antibodies to the same peptide recognize and bind to the same protein, allowing the nucleotide sequences to then undergo complementation. The resulting hybridized DNA tag is then amplified via qPCR.

Levels of protein expression were quantified and converted to a Normalized Protein Expression (NPX) value.

### Genetically modified murine model

Brain endothelial cell conditional *CCM3 (pdcd10)*—null mice (*Slco1c1-CreERT2*; *Pdcd10^fl/fl^*; becKO) were bred by crossing mouse strain with *Slco1c1* promoter-led tamoxifen-activated Cre recombinase (*Slco1c1-CreERT2*) with a mouse strain with loxP-flanked *Pdcd10* (*Pdcd10^fl/fl^*). Tamoxifen was injected via intragastrical injection into becKO mice on postnatal day 1 to induce genetic differentiation (ablation of *CCM3* in brain endothelial cells).

Adult becKO Mice, and age-matched control littermate *Pdcd10^fl/fl^* mice were sacrificed for brain removal. Brains were preserved for sectioning. Please see reference for additional details (Lai et al., 2022).

### Immunohistochemistry—immunofluorescence

Surgically excised cavernoma tissue removed from pediatric CCM patients were fixed in formalin and embedded in blocks of paraffin as part of routine clinical practice, as previously described (Sesen et al., 2024). From these, 8 μm thick tissue sections were cut to prepare slides for staining.

CCM patient tissue sections and previously mentioned becKO and control littermate mice brain sections were subjected to immunohistochemistry staining. Slides were deparaffinated in a series of solutions of xylene, ethanol, and distilled water. The slides were then incubated in Tris-EDTA (pH 9.0) at 95° C for 25 min to perform antigen retrieval and optimize antibody binding.

Slides were washed in phosphate-buffered solution (PBS; #70011–044, Gibco) and incubated in PBST (PBS with 0.1% Triton X100) containing 3% bovine serum albumin (BSA; Sigma Aldrich, #A7906) for 1 h.

LOX-1 antibody (Abcam, #ab126538) was diluted to a working concentration of 5 μg/mL in a solution of PBST containing 1% BSA. This solution was applied to the slide overnight at 4°C. The slides were then washed with PBS and incubated with secondary antibody (anti-rabbit) conjugated with Alexa Fluor 594 (A11012, Invitrogen) for 1 h. Slides were then washed again, with PBS and PBS containing DAPI (#H-1200, Vector Labs).

### Western blotting

CCM tissue surgically removed from pediatric CCM patients was cultured *ex vivo*, with cells isolated as previously described (Sesen et al., 2024). The freshly excised tissue was subjected to collagenase/dispase (Roche; Indianapolis, IN) at 37°C to promote disassociation, pelleted by centrifugation at 1200 RPM for 5 min, and then reconstituted in endothelial basal media, EBM2 (#C3156 Lonza; Walkersville, MD) with 5% FBS (#S11550, R&D Systems; Minneapolis, MN). Cells were plated on plates coated with attachment factor (Gibco; Grand Island, NY). Upon cells reaching near confluence, they were sorted for CD31+ expressing endothelial cells using Invitrogen Dynabeads (Carlsbad, CA). The sorted CD31+ cells were resuspended in EGM2 growth media and plated on plates coated with a gelatin attachment factor (#006–100, Gibco).

Human brain microvascular endothelial cells (HBMVEC) cells were sourced from Cell Systems (Kirkland, WA) and were cultured in EBM2 media (Lonza #CC3156, Walkersville, MD) with EGM2 SingleQuots Supplements (Lonza, #CC4176) containing Hydrocortisone (0.2 mL), hFGF-B (2 mL), VEGF (0.5 mL), R3-IGF-1 (0.5 mL), ascorbic acid (0.5 mL), hEGF (0.5 mL), GA-1000 (0.5 mL), heparin (0.5 mL), Penicillin–Streptomycin (5 mL, Gibco #15140122), Normacin (1 mL, Invivogen #ant-nr-1), and enriched with 10% FBS (50 mL)(all supplements dissolved in in 500 mL total media).

Cell cultures were harvested and lysed with RIPA (#20–188, EMD Millipore; Burlington, MA) with protease inhibitors (#78442, ThermoScientific). Lysates were analyzed for protein content using a Bradford protein assay. Aliquots were made for each lysate to ensure equal protein loading between all samples. These aliquots each received reducing buffer containing beta-2-mercaptoethanol (#J60015.AC, ThermoScientific) and were boiled for 5 min directly prior to loading into a 10-well, 50-μL Bio-Rad gel for electrophoresis. The gel was run at 90 V for 90 min with Bio-Rad running buffer (#1610732). The proteins on the gel were transferred to a PVDF membrane using Bio-Rad Fast Transfer Buffer (#1610734, Bio-Rad) and a Bio-Rad Trans-Blot Turbo Transfer apparatus (#1704150).

The membrane was incubated in Tris-buffered Solution (#J26938.K7, ThermoScientific) with 0.1% Tween20 (TBST) containing 5% non-fat dry milk (protein; #1,706,404, Bio-Rad) for 1 h. Following blocking, the membrane was incubated overnight at 4°C in a solution of TBST containing primary antibody.

Rabbit anti-LOX-1 primary antibody (ab214427, Abcam) and rabbit anti-GAPDH primary antibody (#2118S, Cell Signaling) were used to probe for LOX-1 and GAPDH, respectively. Secondary HRP-conjugated goat anti-rabbit antibody (#31460, ThermoFisher) diluted 1:10,000 in TBST was used to bind to primary antibody.

Femto-strength ECL (#34095, ThermoScientific) was used to develop the LOX-1 probe, while normal-strength ECL (#32106, ThermoScientific) was used to visualize GAPDH. In between probing for the two proteins, the membrane was cleared of antibodies by a 25-min incubation in Restore Stripping Buffer (#21095, ThermoScientific), after which the membrane was washed with TBST and re-blocked before overnight primary antibody incubation.

The anti-LOX-1 primary antibodies and anti-rabbit secondary antibodies were removed from the membrane with (#21059, ThermoScientific) Restore™ Stripping Buffer, and the membrane was re-blocked in TBST containing milk protein.

### Protein analysis

Relative levels of protein were determined by obtaining urinary concentration of the protein of interest (pg/mL) and dividing this value by the total protein concentration (mg/mL) of the same urine sample.

LOX-1 levels were measured by LOX-1 ELISA kits (Abcam, ab212161). Procedures were followed as written by the manufacturer. Samples were run in duplicate. The results were read using a FilterMax F3 spectrophotometer (Molecular Devices). Urine samples were concentrated using ThermoFisher 10 K protein concentrators (#88513, ThermoScientific), with centrifugation at 4°C at 13,000 RPM.

Total protein concentrations were determined by adding 200uL of a 1:5 (Bradford dye: water; #5000006, Bio-Rad Laboratories) to a 10 uL sample of the urine.

LOX-1 urine samples are reported in picograms LOX-1 per milligram total protein (pg/mg).

### Statistical analyses

Mann Whitney U-test was employed to analyze the ELISA data, detailing the LOX-1 presence in pediatric CCM and control patient urine, for statistical significance.

Mann Whitney U-tests were also used to analyze the ImageJ data of immunofluorescence, for both the experiment staining LOX-1 in human CCM tissue and healthy brain artery, and mouse *CCM3 becKO* and age-matched control *Pdcd10^fl/fl^* littermate brain tissue.

A one-sample t-test was performed on the Western Blot data, to analyze statistical significance of the ratio of LOX-1/GAPDH chemiluminescent signal between CCM patient lysates and control HBMVEC lysate wells.

## Results

### Patient demographics—OLINK

A total of 23 CCM patients and 24 control patients (urine samples) were included in this study (Table 1). Additional patient materials were included in this study as CCM patients are frequently admitted and the tissues were collected in accordance with approval from the Boston Children’s Hospital Institutional Review Board.

**Table 1:** Patient demographics for OLINK proximity extension analysis.

Ages y.o.	1	2	3	4	6	7	8	9	11	13	14	15	17	19
Neuro control														
N#	2	3	2	4	1	1	1	1	2	1	1	2	2	1
Sex	Males	Females												
N#	13	12												
Congenital Condition	FF	Chiari												
N#	10	14												
CCM														
Ages y.o.	1	2	3	6	7	8	9	10	12	13	14	16	17	19
N#	1	1	1	2	1	1	1	1	2	2	1	3	3	1
Sex	Males	Females												
N#	11	10												

In Table 2, clinical details of CCM surgical specimens that are used for primary cell culture and tissue analysis is presented.

**Table 2:** Demographics of the pediatric cavernoma patients whose samples were used for histological and protein (western blotting) analysis.

CCM-#	A	B	C	D	E	F	G
Sex	Male	Female	Female	Data censored due to IRB	Female	Male	Male
Age (years)	16	2.5	9	6	5	9	17
Familial	No	Yes (CCM3)	No	Data censored due to IRB	No	No	Yes (CCM2)
Location	Right temporal	Cerebellar	Right Parietotemporal	Data censored due to IRB	Right parietooccipital	Right temporal	Right frontoparietal
Seizure	Yes	Yes	No	Data censored due to IRB	Yes	No	No
Hemorrhage	Yes	No	Yes	Data censored due to IRB	Yes	Yes	No
Multiple Lesions	No	Yes	No	Data censored due to IRB	No	No	Yes

**Figure 1:**
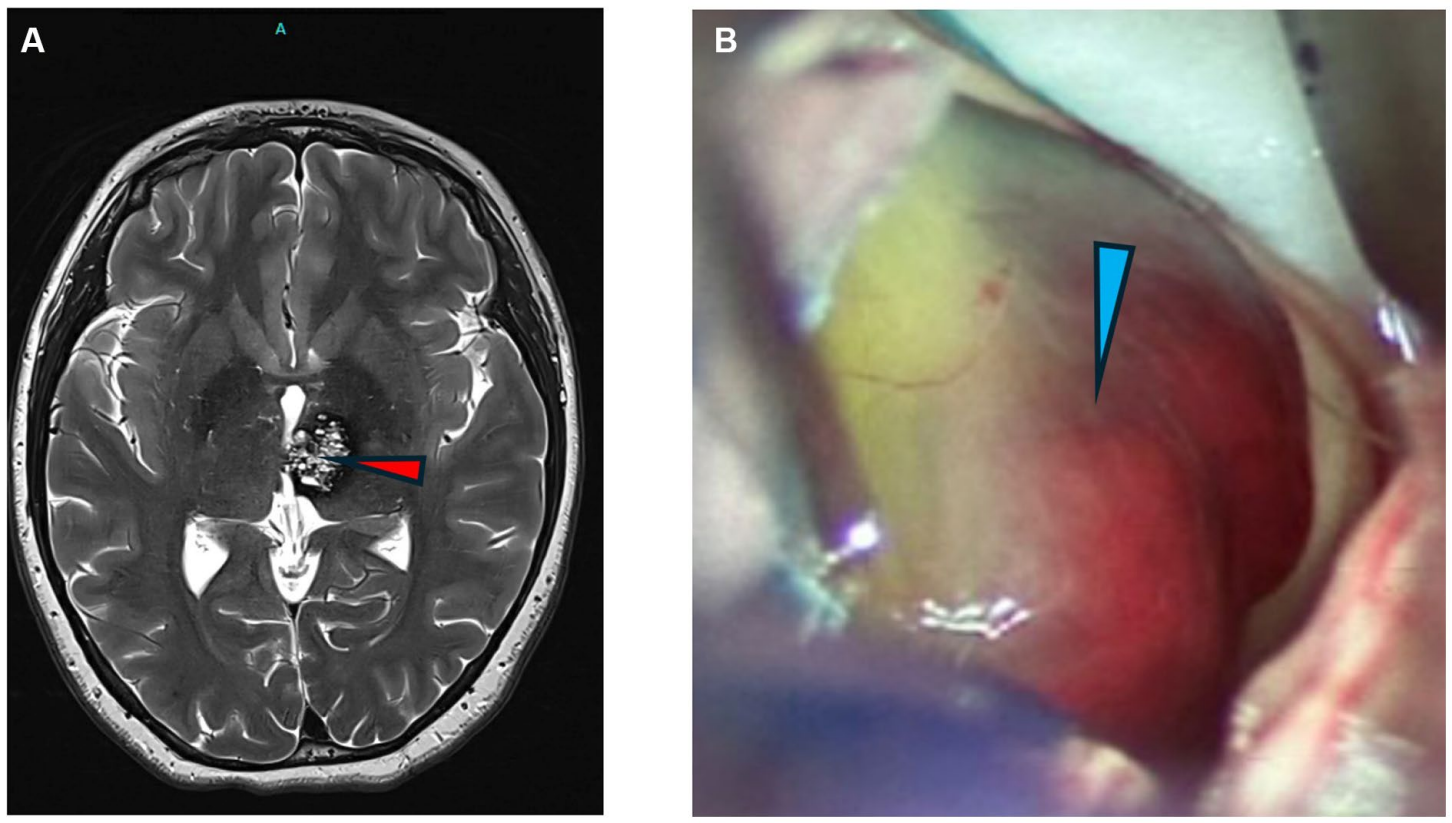
Images of cavernomas. **(A)** Axial T2 weighted MRI of the brain showing a left thalamic cavernous malformation (arrowhead). **(B)** Intraoperative photograph of CCM lesion with hemorrhagic staining of periventricular lesion.

Pediatric cavernoma patients treated surgically had complete removal of the intracranial vascular malformation and pre-and post-operative imaging are shown (Figure 1).

### OLINK

The proximity extension assay analyzed the relative levels of signal for 2,925 proteins between control and CCM patient urine samples. The levels of protein expression were expressed internally as raw numbers, converted to an arbitrary Normalized Protein Expression (NPX) value.

Olink data identified higher or lower concentrations relative to control samples.

**Figure 2:**
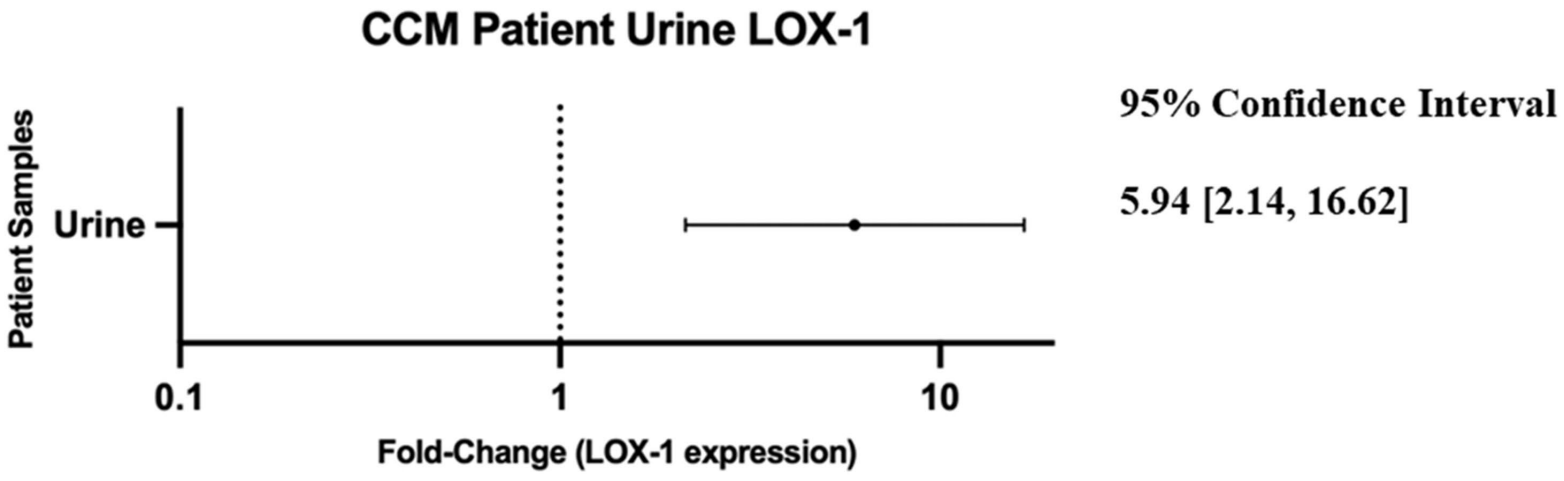
Forest plot of 95% confidence interval range for fold-change difference of normalized relative expression between CCM patient urine and neuro control patient urine as reported by OLINK data. Note: a fold-change of 1 would imply no difference in protein detection between control and cavernoma urine.

Review of the data revealed that LOX-1 displayed the highest disparity between control and CCM urine measured at ~6-fold greater expression in the CCM samples (Figure 2).

Of the 23 CCM urine samples and the 24 control urine samples, one of each fell out of the OLINK internal incubation control acceptance criteria and were excluded from analysis. One protein included in the panel, KNG1, was excluded from analysis due to failure to meet quality control criteria.

### LOX-1 expression in patient urine samples

A LOX-1 ELISA was used to independently determine the concentrations of LOX-1 protein for each of the control and CCM patient urine samples, as normalized with Bradford assay. LOX-1 concentration was divided by total protein concentration to calculate the relative concentration of LOX-1 for each sample. These [LOX-1]/ [total protein] values, reported in picograms per milligram of protein, were analyzed with a Mann Whitney U test (Duggins-Warf et al., 2023).

**Figure 3:**
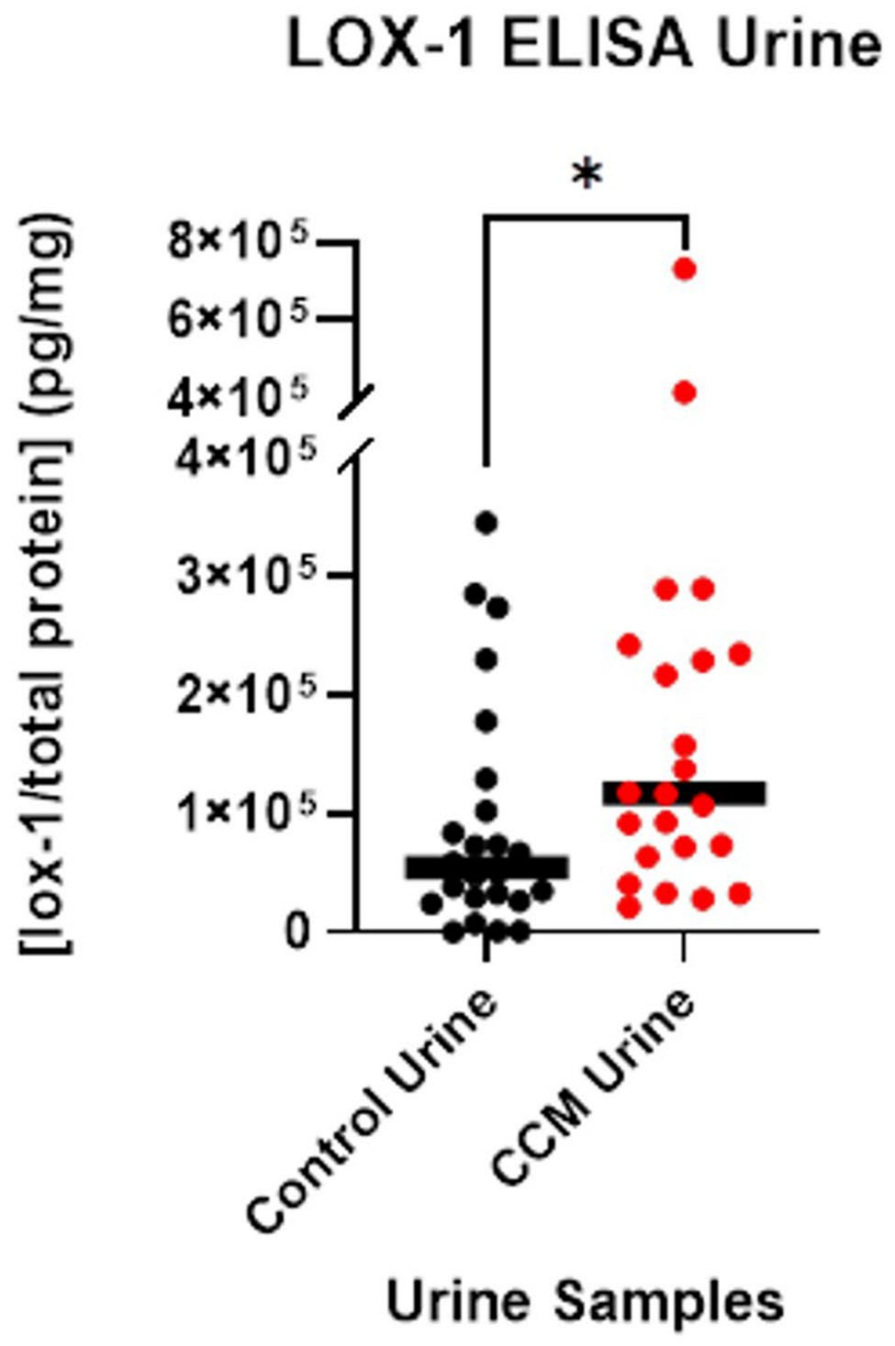
LOX-1 expression in urine. LOX-1 expression was quantified using ELISA on urine samples from CCM patients and matched controls.

The average LOX-1 level in CCM urine was 166,960 pg/mg as detected by ELISA, while control urine was 91,410 pg/mg, a ~ 2-fold difference between CCM and control samples. Statistical analysis with Mann Whitney U-test revealed a significant difference in these values between control and CCM urine (*p*-value = 0.027). These results, as shown in Figure 3, demonstrate elevated LOX-1 levels in the urine of cavernoma patients relative to matched controls. LOX-1 has been demonstrated to induce oxidative stress, and in turn, oxidative stress stimulates LOX-1 expression in a positive feedback manner (Dai et al., 2016). In particular, the overexpression of NADPH oxidase, Mitochondrial Reactive Oxygen Species (mtROS) generation and activation of redox-sensitive signals (MAPKs and NF-κB) all are markers of LOX-1 activation (Li et al., 2003; Dai et al., 2016).

Given that both oxidative stress and the upregulation of NADPH oxidase, mtROS generation and redox-sensitive signals have been previously associated with CCM disease pathogenesis, as reported in original articles (Goitre et al., 2014, 2017; Marchi et al., 2015; Retta and Glading, 2016; Antognelli et al., 2018a,b; Perrelli and Retta, 2021; Perrelli et al., 2023), we analyzed the levels of proteins associated with NADPH oxidase and mtROS activation (Supplementary Table 1). We found that only urinary Adrenodoxin mitochondrial and NADH dehydrogenase [ubiquinone] iron–sulfur protein 6 mitochondrial to be significantly different between CCM and controls.

LOX-1 is a scavenger receptor for oxidized low density lipoprotein receptor 1 (ox-LDL) which plays an important role in the development of atherosclerosis (Xu et al., 2013), we analyzed the plasma levels of ox-LDL and found it to be significantly higher in plasma of CCM patients vs. controls.

Galectin-3 has recently been identified as a novel potential prognostic/predictive biomarker and therapeutic target for CCM disease (Kar et al., 2024). There is evidence that it can enhance endothelial LOX-1 expression and promote endothelial dysfunction by inducing inflammation via the LOX-1/ROS/p38/NF-κB-mediated signaling pathway (Ou et al., 2019). However, we found that the urinary galectin levels were not significantly different between CCM and controls (Supplementary Table 1).

### LOX-1 expression in cell lines

**Figure 4:**
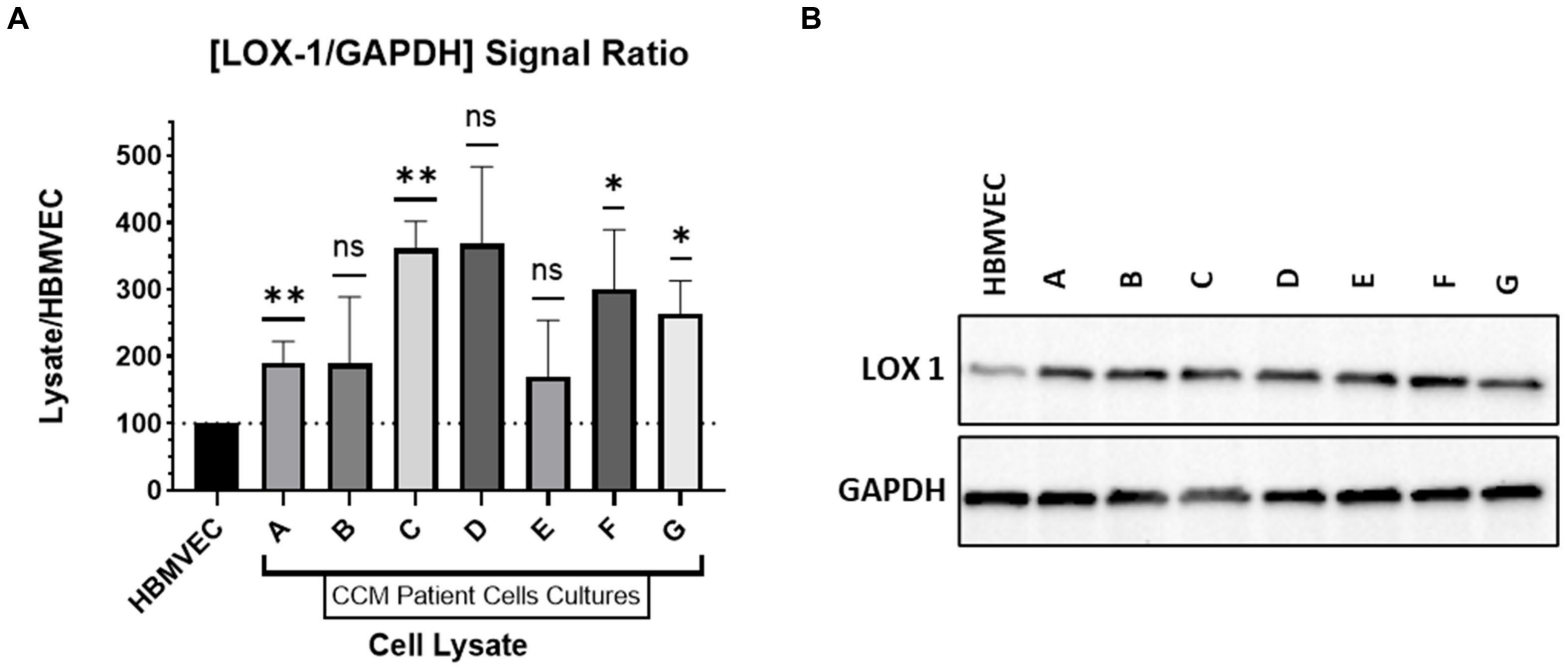
Western Blotting. **(A)** Relative expression of LOX-1 in cultured CCM lysates compared to HBMVEC. **(B)** Western Blot of LOX-1 expression with HBMVEC and CCM lysates.

Cell cultures derived from CCM patients were profiled for endothelial cell characteristics using endothelial specific markers, confirming that the primary cell culture contained solely endothelial cells (Sesen et al., 2024). Lysates of patient CCM cells and of HBMVECs were run via gel electrophoresis to determine LOX-1 and GAPDH expression. Probing was done with incubation with rabbit anti-LOX1 (ab214427) and anti-GAPDH. The signal strength of the chemiluminescent bands was analyzed with ImageJ for LOX-1 and GAPDH probes. The signal strength of LOX-1 was compared to that of GAPDH for each band to generate a signal ratio. The signal ratio for HBMVEC was normalized to have a value of 100%. The signal ratio for each CCM lysate was then compared against that of the normalized HBMVEC ratio. Figure 4 contains this probing signal data. The western blotting was repeated (*n* = 3) for all lysates except CCM Patient Lysate D, as the number of cells available were limited.

CCM lysates were compared against HBMVEC lysate to determine relative LOX-1 levels. As demonstrated in Figure 4, LOX-1 has greater expression in CCM-derived cell lines relative to HBMVEC. We demonstrate significant elevations of LOX-1 expression in CCM-derived cell lines relative to control HBMVEC cells.

### Immunohistochemistry—fluorescence—patient cavernoma tissue

Control slide tissue was comprised of an endothelial cell-enriched cutout of an artery, to examine the population of normal cells most closely related to the pathologic tissue in CCM. Excitation of conjugated Alexa Fluor 594 dye causes emission of red light that signals presence of the protein of interest, LOX-1. Exposure settings were optimized to eliminate background signal. Staining of CCM tissue slides reveals significant differences in LOX-1 presence in the disease tissue compared to control slides of endothelial cells in blood vessels from the brain of individuals without CCM, with Control slides demonstrating only background levels of signal, while CCM tissues exhibited increased LOX-1 expression. Four fluorescence images were taken from healthy adult brain artery imaging, and three fluorescence images were taken, each, from slides of the 7 CCM pediatric brain tissues (CCM patients A-G, as described in Table 2), for a total of 21 CCM data points.

**Figure 5:**
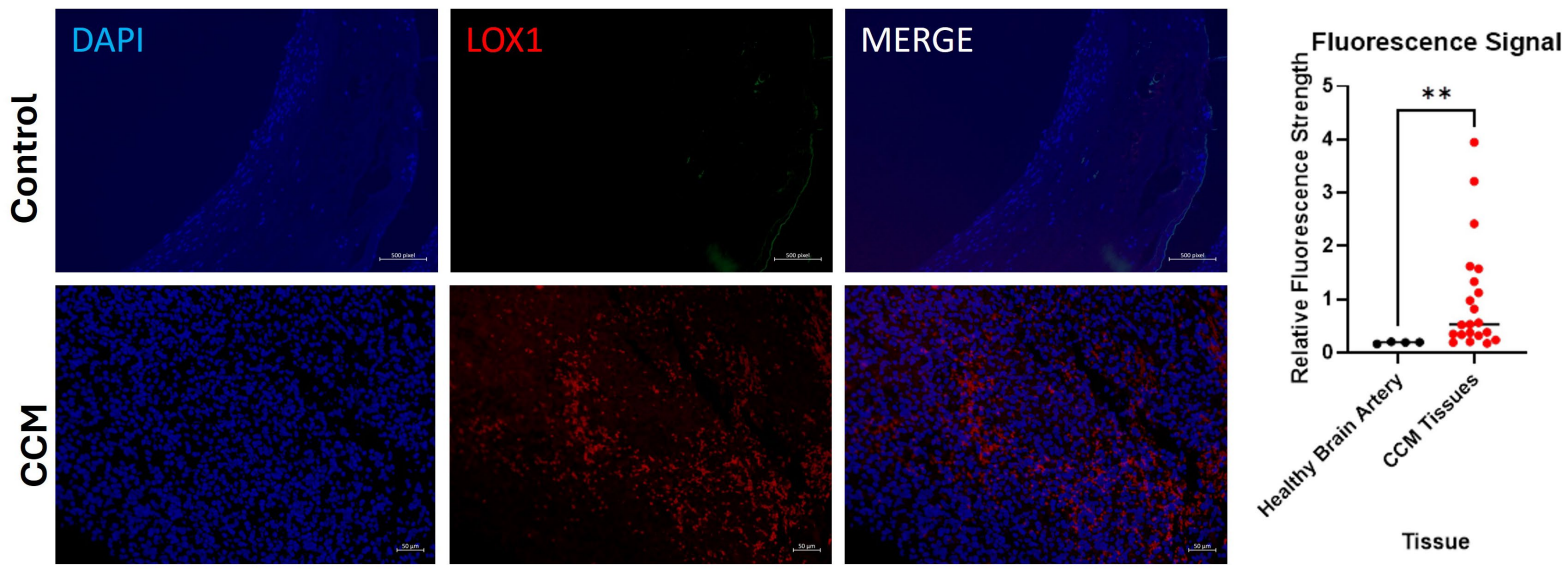
Fluorescence images of disease-state CCM tissue. Tissue samples were originally excised from pediatric patients who had underwent surgical removal of cavernoma tissue. DAPI staining of cell nuclei in blue; Alexa Fluor 594 (red) staining of LOX-1. Comparison of signal intensity of LOX-1 related fluorescence in CCM tissue compared to control healthy brain artery, with 5.58-fold increase of overall expression in CCM. The biological and technical replicate for control brain artery staining was limited due to availability. Three fluorescence images were taken from each of the 7 patients (CCM patients A-G).

Quantitative fluorescence signal analysis conducted on the slides with ImageJ corroborated this visual finding. AF 594 signal increased in the CCM as compared to control (Figure 5). Relative fluorescence intensity for patient CCM Tissues (1.04) was on average 5.58 times greater than that for controls (0.187). A Mann Whitney U-test performed on these data revealed a statistically significant increase in fluorescence in CCM patient tissue compared to the healthy control (*p*-value = 0.0042).

### Immunohistochemistry—fluorescence—mouse brain tissue

**Figure 6:**
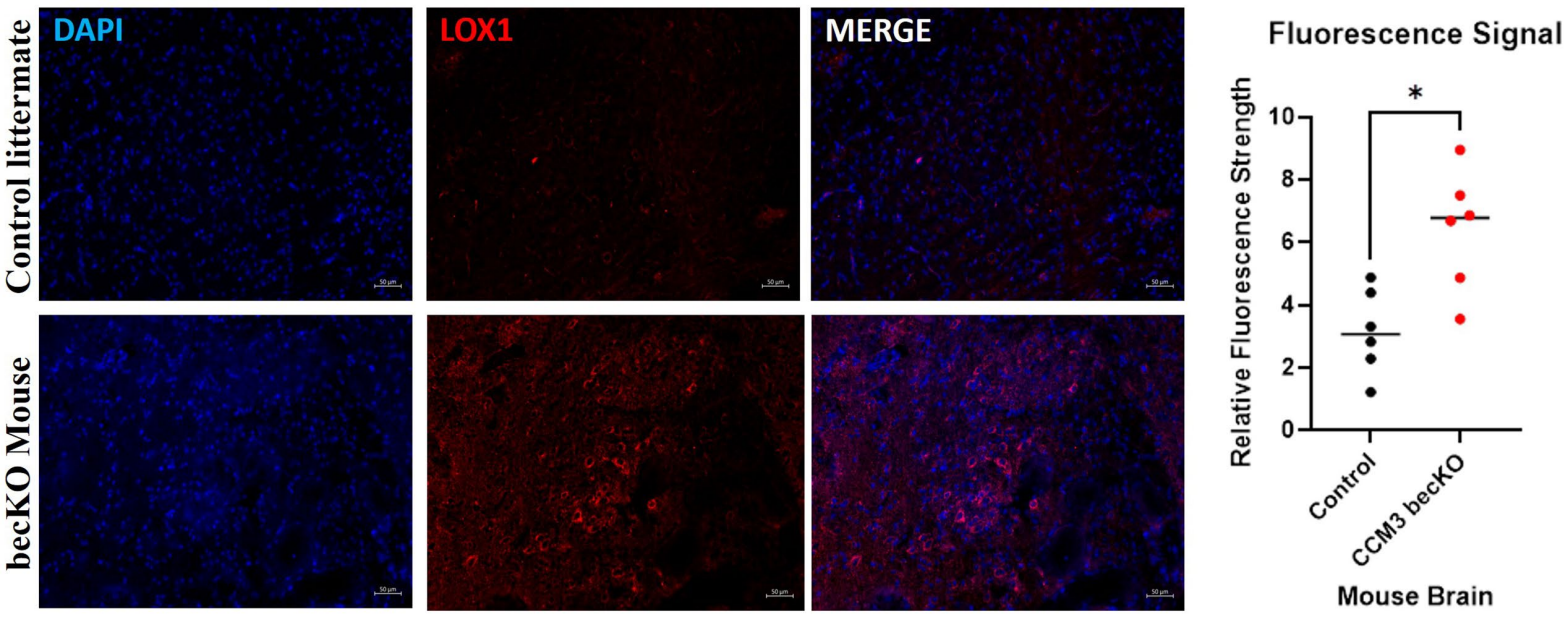
Fluorescence images of age-matched control littermate *Pdcd10^fl/fl^* mice and *CCM3* becKO mice. DAPI staining of cell nuclei in blue; Alexa Fluor 594 (red) staining of LOX-1. Comparison of signal intensity of LOX-1 related fluorescence in *CCM3* becKO mice and age-matched control littermate *Pdcd10^fl/fl^*, with 2.03-fold higher intensity in *CCM3* becKO mice.

Brains of *CCM3* becKO mice and control littermate *Pdcd10^fl/fl^* mice were sectioned and stained for LOX-1. Excitation of conjugated Alexa Fluor 594 dye causes emission of red light that signals presence of the protein of interest, LOX-1. Exposure settings were optimized to eliminate background signal. Fluorescence staining revealed large visual differences indicating a significant disparity in LOX-1 presence between the brain tissues of the two strains of mice, seen in Figure 6. LOX-1 staining revealed much higher levels of protein in the becKO mouse compared to controls. Relative fluorescence intensity for becKO tissue (6.41) was on average 2.03 times greater than that for controls (3.15). A Mann Whitney U-test performed on these data revealed a statistically significant increase in fluorescence in the *CCM3 becKO* mouse brain tissue compared to brains of age-matched control *Pdcd10^fl/fl^ littermates* (*p*-value = 0.0152).

### CCM patient genetic sequencing

Whole exome sequencing was conducted on the CCM patient samples that were used for histological and western blot analysis (Sesen et al., 2024). Sequencing revealed no evident mutations in the LOX-1 gene for any of the patients. Sequencing did reveal individual point mutations for CCM2 and CCM3 genes, respectively, for CCM patients G and B, indicating the familial form of CCM for these patients. These latter results are displayed in Table 2.

## Discussion

### CCM background and utility of urinary biomarkers

As reviewed in the introduction, CCMs are pathologic structures comprised of dysfunctional capillaries prone to growth and bleeding (Smith, 2024). While significant progress has been made in discovering specific mutations that contribute to the pathophysiology of these lesions, there remains a pressing clinical need to develop better tools to identify the presence of CCM in at-risk individuals, while also improving the follow-up of known disease (Revencu and Vikkula, 2006; Smith, 2024). The current “gold standard” of MRI provides excellent visualization of CCMs, but has limitations, including high cost, availability restrictions (with a finite number of scanners at specialized centers) and the risk of anesthesia often needed to facilitate imaging with children (Salerno et al., 2018). The impact of these limitations become compounded and increasingly evident over time with CCM, given the long-term nature of the disease, with many individuals requiring lifetime monitoring. The use of non-invasive biomarkers, such as urine sampling to measure levels of specific molecules, has the potential to complement the use of MRI by offering cheap, easy and frequent testing (that can be mailed, obviating the need for travel in many cases) and which provides a metric (quantifiable protein levels) which is distinct from the imaging data derived from MRI (Duggins-Warf et al., 2023; Sequeira-Antunes and Ferreira, 2023).

Biomarkers, and specifically urinary biomarkers, have clear precedents for utility and value in the management of a wide range of medical conditions, with one of the most widely known being human chorionic gonadotropin (hCG) levels used as a biomarker for pregnancy (Peris et al., 2024). Specific to disease in the central nervous system, urinary biomarkers have been successfully demonstrated to detect, diagnose and follow the response to therapy of brain tumors, stroke conditions such as moyamoya and cerebrovascular lesions, including arteriovenous malformations (AVM) and—recently from our laboratory—CCMs (Smith et al., 2008; Akino et al., 2014; Smith, 2015; Fehnel et al., 2020; Sesen et al., 2021a, b, 2024; Lehman et al., 2022). The development of these biomarkers has been an exciting advance, with a recent national multicenter trial demonstrating their potential utility in longer term follow up of CNS disease and these results have spurred interest in the development of additional markers for other conditions (Baxter et al., 2020).

### Methodology and rationale for biomarker selection

There are numerous strategies for the discovery of disease-specific biomarkers. In this study, we sought to leverage two complementary approaches, first performing a high-throughput analysis comparing a cohort of samples from affected CCM patients to matched controls. Then subsequent validation using patient-derived samples incorporating source tissue as well as biospecimens from outside the CNS (including urine), demonstrating concordant expression between the presumed source of the biomarker (the CCM cells) and the downstream sampling medium (the urine). Using this methodology, which we have successfully employed in other work, we identified the molecule LOX-1 as a putative CCM-related biomarker.

Importantly, LOX-1 (lectin-type oxidized LDL receptor 1), a 50 kDa transmembrane protein, has biological functions that make it a plausible candidate as biomarker for CCM. LOX-1 has been implicated in endothelial cell dysfunction and ischemia (Chen et al., 2002; Barreto et al., 2021). There is data to show that endothelial cells that are experiencing ischemia or transmural shear force (as occurs in CCM) increase expression of LOX-1 (Barreto et al., 2021). In combination, this prior literature provides a rationale as to why LOX-1 may be elevated in patients with CCM, and we hypothesize that LOX-1 overexpression is a secondary effect of the ischemia and altered cerebrovascular hemodynamics that are well-documented in CCM.

It is worth noting some biomarkers may show marked variability with individual patients, and related work has demonstrated that in some cases this variability can be associated with key pathophysiological features (such as tumor size in tumor biomarkers). Consequently, while it is important not to read too much into this initial study, subsequent work with larger populations may be able to provide better statistical power in order to assess for these sorts of associations with LOX-1 and CCM.

### Demonstration of increased LOX-1 expression in CCM patients, validation of screening methodology and linking source tissue to downstream sampling

Our initial experiment involved the use of a general screening analysis comparing the disease cohort to a set of matched control individuals, using the OLINK assay—a commercially available service that is designed to be extremely sensitive to the discovery of differential expression by augmenting detection of selected molecules. This assay revealed significantly increased expression of urinary levels of LOX-1 in CCM patients as compared to age- and sex-matched control patient samples (Figure 2). While these results were encouraging, we subsequently performed a validation of the data using ELISA, which independently corroborated the differential expression of LOX-1 in CCM patient samples. As seen in Figure 3, analysis by Mann–Whitney U test revealed that CCM patient urine showed a statistically significant amount difference in LOX-1 compared to control urine.

It is worth noting that there is a difference in the relative increase of LOX-1 levels as reported by the OLINK analysis (~6 fold) and the ELISA (~2 fold). It is likely that these differences are due to the technical differences in the methodologies employed. OLINK relies on both protein–protein interaction (similar to ELISA) and polymerase chain-reaction, with the technology specifically designed to purposefully highlight the differences in protein quantity between samples. This technique is—by design—highly sensitive and, as it was in this study, is meant to help discover potential differential expression patterns. To be sure that this difference was not solely due to the technical aspects of OLINK, we performed the ELISA experiments, which—even without the “magnification effect” of OLINK—still confirmed a significant difference in LOX-1 expression.

Demonstrating that this same differential expression was present in both a third-party commercial analysis and with our own laboratory experiments substantiated the validity of the findings. Following these two analyses, we then sought to emulate work done by our laboratory and others that tests the hypothesis that the pathologic tissue (CCM) is the putative source of the biomarker found in the downstream sampling (urine). In pursuit of this goal, we obtained primary cell lines derived from surgically resected CCM tissue, which had been confirmed by neuropathology. These CCM-derived endothelial cell lines, developed as previously described, were compared to control brain-derived endothelial cells (HBMVECs), and clearly demonstrated increased expression of LOX-1 relative to the controls (Figure 4; Sesen et al., 2024). Finally, to control for the unlikely but possible chance that LOX-1 expression may have been somehow dysregulated by creating the cell lines, we also performed immunofluorescent staining on pathology slides directly—and this successfully revealed marked LOX-1 expression in the tissue of origin, in addition to demonstrating greater relative LOX-1 expression in the CCM tissue when compared to control, non-CCM brain vascular tissue. In aggregate, these data provide support for the hypothesis that the CCM, and specifically the dysfunctional endothelial cells, serves as a source of increased LOX-1 expression.

Oxidative stress and high NADPH oxidase and mtROS generation have been previously associated with CCM (Goitre et al., 2014, 2017; Marchi et al., 2015; Retta and Glading, 2016; Antognelli et al., 2018a,b; Perrelli and Retta, 2021; Perrelli et al., 2023). LOX-1 has been demonstrated to induce oxidative stress, and in turn, oxidative stress stimulates LOX-1 expression in a positive feedback manner (Dai et al., 2016). However, in our cohort, most of the proteins involved in the oxidative stress-NADPH-mtROS axes were not significantly different between CCM and controls (Supplementary Table 1). While the oxidative stress-NADPH-mtROS axes are established to play a role in the CCM pathogenesis, proteins involved in those axes might not be released into urine to serve as a biomarker.

As oxidative stress accompanies atherogenesis and several other vascular disease states, it is also well-known that LOX-1 is a scavenger receptor for ox-LDL and plays an important role in the development of atherosclerosis and its sequelae (Xu et al., 2013). In this light, we investigated whether if there are any correlation between urinary levels of LOX-1 and plasma levels of ox-LDL in the CCM cohort of CCM. This was performed as it has been shown that CCM disease has been linked to an increased risk for atherosclerosis (Sesen et al., 2019). However, we found ox-LDL to be also significantly expressed in the plasma of CCM patients (Supplementary Table 2) indicating that higher LOX-1 levels in CCM patients might correlates with increased risk for atherosclerosis and vascular disorders. While this study offers an interesting lead, this possible correlation needs to be studied in more details in future comprehensive research.

### Potential impact of mutations

In this study, it is important to acknowledge that CCMs may be heterogeneous in their genetic and mutational profiles. As described in the result section, the patient samples were subjected to genetic analysis examining for known CCM mutations, and there were no obvious biased results (One patient had a CCM2 mutation, one had a CCM3 mutation, and the other three had no known germline mutations). While data from five patients is too small for statistical analysis, this data suggests that the LOX-1 results are likely generalizable, at least within the limitations of this small sample size. In addition to examining CCM mutations, we also performed analysis of the LOX-1 gene, to investigate whether the elevated levels may be a result of a LOX-1 mutation. The analysis revealed normal LOX-1 genes in the cell lines, supporting our hypothesis that LOX-1 overexpression in CCM is a secondary, downstream effect of the underlying pathology—as seen in other disease states—and not due to a unique LOX-1 mutation found in our cohort.

### Limitations and future directions

There are several limitations related to this study. First is the relatively small number of samples, which can affect the power of the analysis and the generalizability of the results. Despite the small numbers, we attempted to be stringent with our analysis, presenting both internal (from our laboratory) and external (from OLINK) independent experiments, along with expanding our work to include primary source tissue and genetic studies. We are currently working on expanding this work with a possible multicenter collaboration, with consent process and homogenization of sample collection protocols presenting logistical challenges. Importantly, we have been able to successfully carry out multicenter biomarker studies, with presentation of this sort of initial promising data useful for encouraging future recruitment (Baxter et al., 2020).

A second potential limitation is the lack of specificity of LOX-1 as a biomarker. We hypothesize that LOX-1 overexpression is secondary to the ischemia and abnormal hemodynamics of CCM -and therefore not specific to CCM. As such, LOX-1 may be a sensitive—but not specific—marker of disease. However, this lack of specificity may not be relevant to many of the most important potential functions of a CCM biomarker, which center on response to treatment or change over time. There is precedent for the utility of these sorts of general biomarkers in other conditions, such as c-reactive protein (CRP) in monitoring infection, and there is also precedent in increasing the specificity of a general biomarker by combining it with other biomarkers (multiplexing; Smith et al., 2008; Fehnel et al., 2020; Duggins-Warf et al., 2023; Pastorello et al., 2023). These present opportunities for future study, including longitudinal monitoring of LOX-1 levels in CCM patients (including pre- and post-treatment measurements) and multiplexing with other relevant CCM and cerebrovascular-related biomarkers (Smith et al., 2008; Fehnel et al., 2020; Duggins-Warf et al., 2023; Sesen et al., 2024).

Lastly is the question of urine as a sampling medium. While we have focused on the use of urine for the reasons outlined in the manuscript, it would be interesting to assess LOX-1 levels elsewhere, such as cerebrospinal fluid (CSF), stool, or serum. Collaboration with organizations with pre-existing biorepository stores may be an avenue forward to pursue this goal.

## Conclusion

Here we present the first report of elevated levels of LOX-1 in the urine, primary cell lines and surgical specimens of CCM patients relative to matched healthy control subjects. These findings suggest that LOX-1 may have potential utility as a novel target for CCM treatment and implicate a possible role for LOX-1 in the pathogenesis of CCM.

## Figures and Tables

**Table:** 

AVM	Arterio-venous malformation
becKO	Brain endothelial knock out
BSA	Bovine serum albumin
CCM	Cerebral Cavernous Malformations
CNS	Central nervous system
CRP	C-reactive protein
CSF	Cerebrospinal fluid
EDTA	Ethylenediaminetetraacetic acid
ELISA	Enzyme linked immunosorbent assay
FBS	Fetal bovine serum
GAPDH	glyceraldehyde 3 phosphate dehydrogenase
HBMVEC	Human brain microvascular endothelial cell
hCG	human chorionic gonadotropin
HRP	Horseradish peroxidase
IRB	Institutional review board
kDa	Kilodalton
LDL	Low-density lipoprotein
LOX-1	Lectin-like Oxidized low-density Lipoprotein (LDL) receptor-1
MRI	Magnetic resonance imaging
mtROS	Mitochondrial reactive oxygen species
NPX	Normalized protein expression
PBST	Phosphate-buffered saline with Tween
*Pdcd10*	Programmed cell death 10 (gene; also known as *CCM3*)
PEA	Proximity extension assay
qPCR	Quantitative polymerase chain reaction
*Slco1c1*	Solute carrier organic anion transporter family member 1C1
TBST	Tris-Buffered Saline with Tween
VEGF	Vascular endothelial growth factor

## Data Availability

The raw data supporting the conclusions of this article will be made available by the authors, without undue reservation.
