# Supplementary figures and images for: Lectin-type oxidized LDL receptor-1 as a potential therapeutic target for cerebral cavernous malformations treatment
Source: Front Neurosci. 2024 Aug 21;18:1442110. doi: 10.3389/fnins.2024.1442110 (PMC11371587; doi:10.3389/fnins.2024.1442110)

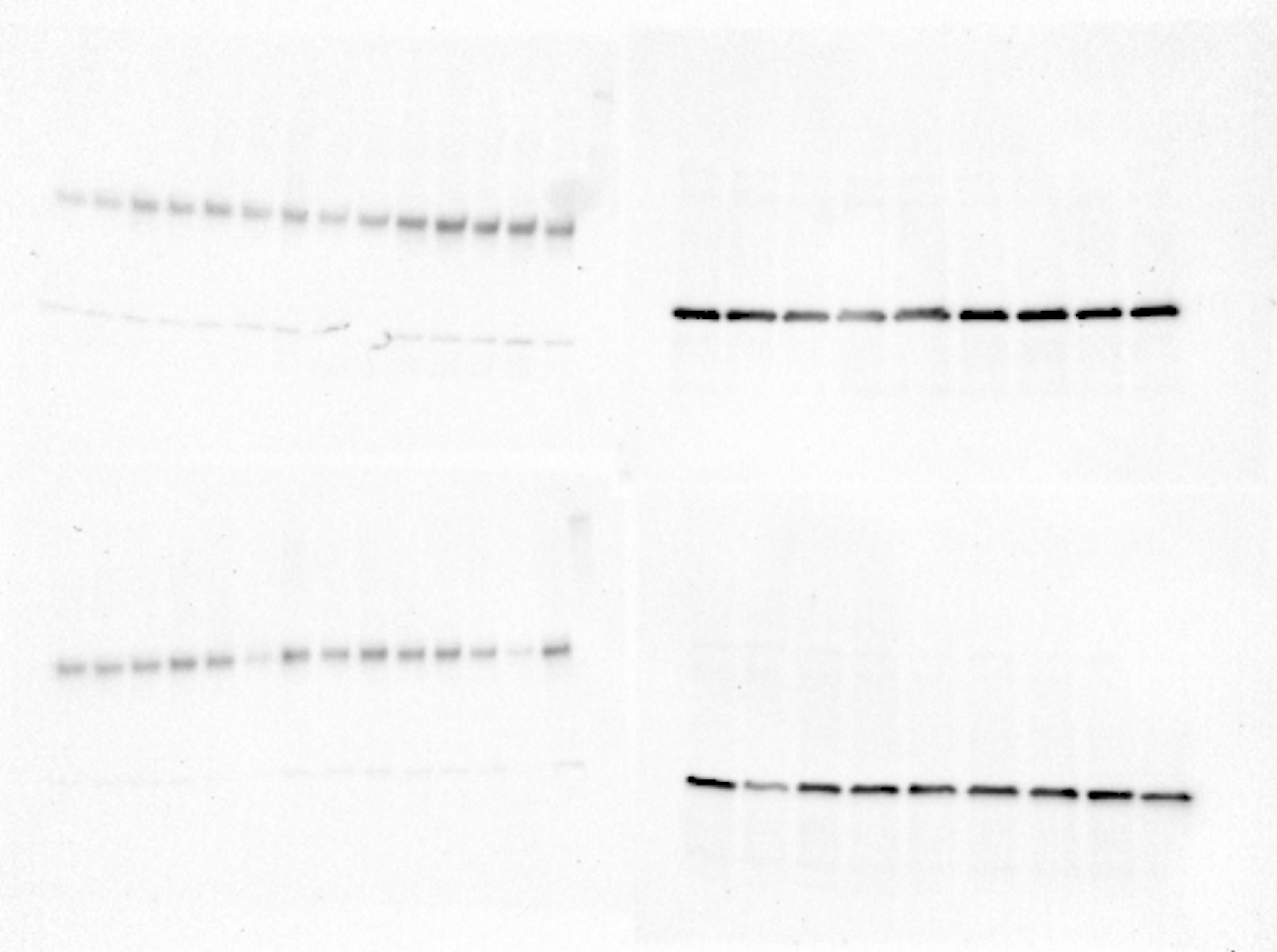

Supplement: Supplementary file 1 [file Image_1.TIF]

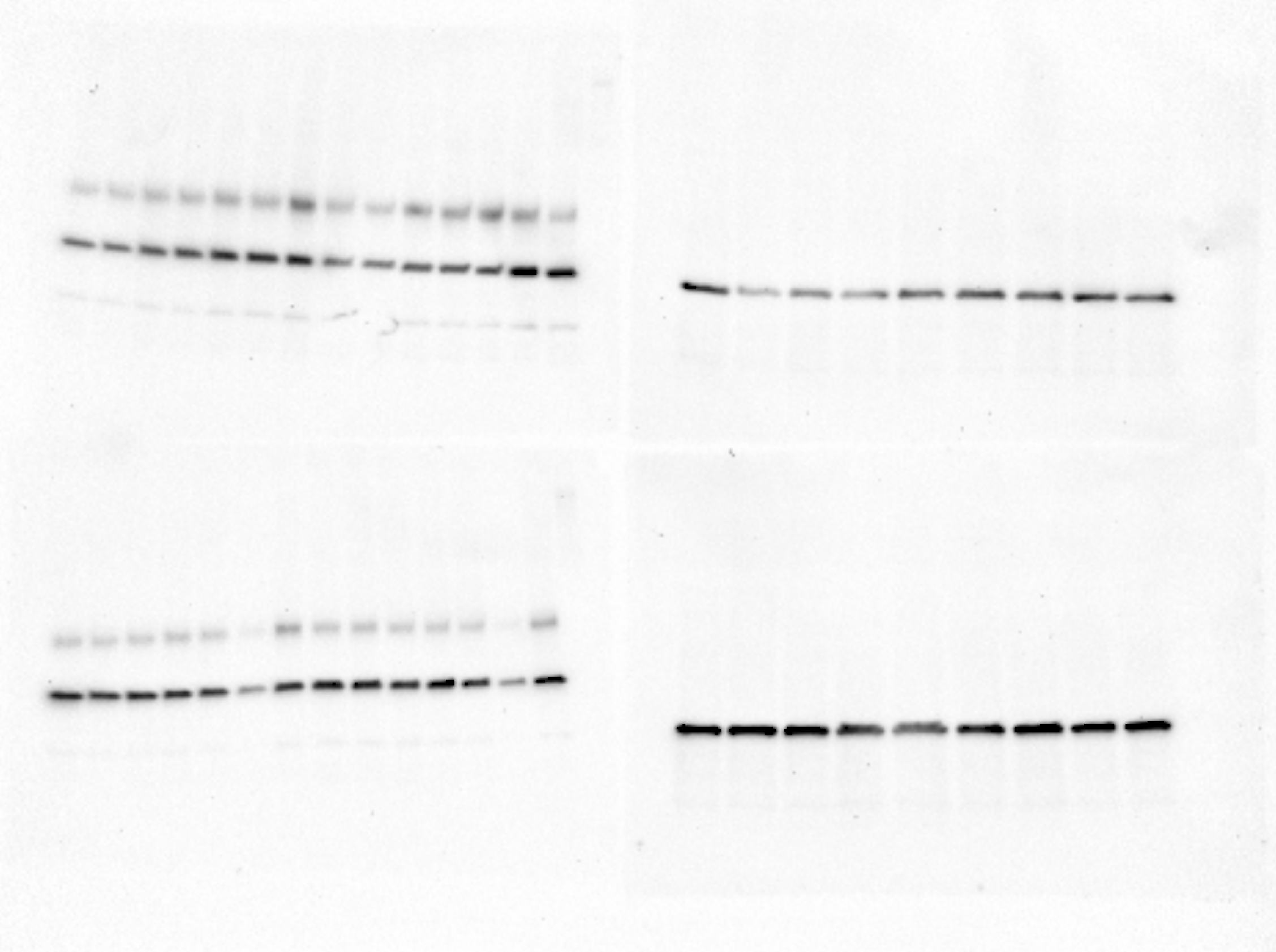

Supplement: Supplementary file 2 [file Image_2.TIF]
