# Supplementary material for: Lectin-type oxidized LDL receptor-1 as a potential therapeutic target for cerebral cavernous malformations treatment
Source: Front Neurosci. 2024 Aug 21;18:1442110. doi: 10.3389/fnins.2024.1442110 (PMC11371587; doi:10.3389/fnins.2024.1442110)
Supplement: Supplementary file 3 [file Table_1.DOCX]

| **Gene** | **Gene Full Name** | **Estimate** | **Fold Change** | **conf.low** | **conf. high** | **Adjusted_p value** | **Threshold** |
| --- | --- | --- | --- | --- | --- | --- | --- |
| ADH1B | All-trans-retinol dehydrogenase [NAD(+)] ADH1B | -0.077207 | 0.94789 | -0.276588 | 0.122173541 | 0.437949098 | Non-significant |
| ADH4 | All-trans-retinol dehydrogenase [NAD(+)] ADH4 | -0.053256 | 0.96376 | -0.194783 | 0.088270991 | 0.450899159 | Non-significant |
| ALDH3A1 | Aldehyde dehydrogenase, dimeric NADP-preferring | -0.110617 | 0.92619 | -0.552465 | 0.33122966 | 0.615215361 | Non-significant |
| CYB5R2 | NADH-cytochrome b5 reductase 2 | 0.4794533 | 1.39422 | -0.773187 | 1.732093845 | 0.443226106 | Non-significant |
| DUOX2 | Dual oxidase 2 | -0.007822 | 0.99459 | -0.268027 | 0.252382492 | 0.951792187 | Non-significant |
| FDX1 | Adrenodoxin, mitochondrial | 1.0261483 | 2.03658 | 0.1263553 | 1.925941309 | 0.026496119 | Significant |
| FDX2 | Ferredoxin-2, mitochondrial | 0.1036719 | 1.0745 | -0.432103 | 0.639446761 | 0.697454541 | Non-significant |
| GPD1 | Glycerol-3-phosphate dehydrogenase [NAD(+)], cytoplasmic | 0.1180051 | 1.08523 | -0.287124 | 0.523134432 | 0.558908351 | Non-significant |
| NADK | NAD kinase | 0.0573404 | 1.04055 | -0.17164 | 0.286320434 | 0.615124736 | Non-significant |
| NCF2 | Neutrophil cytosol factor 2 | 0.0828846 | 1.05913 | -0.484218 | 0.649987506 | 0.768937026 | Non-significant |
| NDUFA5 | NADH dehydrogenase [ubiquinone] 1 alpha subcomplex assembly factor 2 | 0.0274808 | 1.01923 | -0.18861 | 0.243571996 | 0.79822295 | Non-significant |
| NDUFB7 | NADH dehydrogenase [ubiquinone] 1 beta subcomplex subunit 7 | -0.042129 | 0.97122 | -0.178748 | 0.094490647 | 0.536189902 | Non-significant |
| NDUFS6 | NADH dehydrogenase [ubiquinone] iron-sulfur protein 6, mitochondrial | 0.553886 | 1.46803 | 0.0146323 | 1.093139634 | 0.044386827 | Significant |
| SIRT1 | NAD-dependent protein deacetylase sirtuin-1 | 0.01041 | 1.00724 | -0.241813 | 0.262633025 | 0.933850615 | Non-significant |
| SIRT2 | NAD-dependent protein deacetylase sirtuin-2 | -0.34691 | 0.78627 | -1.017944 | 0.324123681 | 0.301910341 | Non-significant |
| SIRT5 | NAD-dependent protein deacylase sirtuin-5, mitochondrial | -0.099592 | 0.9333 | -0.270466 | 0.071281057 | 0.245372706 | Non-significant |
| CD36 | Platelet glycoprotein 4 | -0.001365 | 0.99905 | -0.148112 | 0.145380761 | 0.985069782 | Non-significant |
| LDLR | Low-density lipoprotein receptor | 0.6345791 | 1.55248 | 0.077052 | 1.192106134 | 0.026773396 | Significant |
| LDLRAP1 | Low density lipoprotein receptor adapter protein 1 | 0.0517874 | 1.03655 | -0.257352 | 0.36092678 | 0.736379876 | Non-significant |
| LPA | Apolipoprotein(a) | 0.8098538 | 1.75303 | -0.138131 | 1.757838285 | 0.091850834 | Non-significant |
| LPCAT2 | Lysophosphatidylcholine acyltransferase 2 | -0.113082 | 0.92461 | -0.41154 | 0.185376688 | 0.447812491 | Non-significant |
| LPL | Lipoprotein lipase | 0.3292968 | 1.2564 | -0.499402 | 1.157995229 | 0.426155379 | Non-significant |
| LRP1 | Prolow-density lipoprotein receptor-related protein 1 | 0.0921475 | 1.06596 | -0.420358 | 0.604653257 | 0.717889001 | Non-significant |
| LRP11 | Low-density lipoprotein receptor-related protein 11 | 0.4761717 | 1.39105 | -0.232107 | 1.184450373 | 0.18153448 | Non-significant |
| LRP2 | Low-density lipoprotein receptor-related protein 2 | 0.4311089 | 1.34827 | 0.0615691 | 0.800648671 | 0.023420185 | Significant |
| LRP2BP | LRP2-binding protein | -0.061073 | 0.95855 | -0.260406 | 0.138261388 | 0.538802003 | Non-significant |
| LRPAP1 | Alpha-2-macroglobulin receptor-associated protein | 0.9772346 | 1.96869 | 0.1296604 | 1.824808889 | 0.024978993 | Significant |
| LRRC25 | Leucine-rich repeat-containing protein 25 | 0.1871009 | 1.13847 | -0.471025 | 0.845226406 | 0.568330756 | Non-significant |
| OLR1 | Oxidized low-density lipoprotein receptor 1 | 2.5751543 | 5.95935 | 1.0954986 | 4.054810021 | 0.001128375 | Significant |
| LGALS3 | Galectin-3 | 0.773103 | 1.70894 | -0.034492 | 1.580697679 | 0.060088093 | Non-significant |
| LGALS3BP | Galectin-3-binding protein | 0.9647936 | 1.95178 | -0.367703 | 2.297290429 | 0.15093968 | Non-significant |
| NFKB1 | Nuclear factor NF-kappa-B p105 subunit | 0.3349285 | 1.26131 | -0.046887 | 0.716743709 | 0.083774822 | Non-significant |
| NFKB2 | Nuclear factor NF-kappa-B p100 subunit | 0.0021555 | 1.0015 | -0.138807 | 0.143117538 | 0.975467294 | Non-significant |
| MAP2K1 | Dual specificity mitogen-activated protein kinase kinase 1 | -0.082581 | 0.94437 | -0.224223 | 0.059060158 | 0.245222873 | Non-significant |
| MAP2K6 | Dual specificity mitogen-activated protein kinase kinase 6 | -0.113381 | 0.92442 | -0.39037 | 0.163607993 | 0.412474784 | Non-significant |
| MAP3K5 | Mitogen-activated protein kinase kinase kinase 5 | -0.09877 | 0.93383 | -0.369025 | 0.171484592 | 0.463933458 | Non-significant |
| MAP4K5 | Mitogen-activated protein kinase kinase kinase kinase 5 | -0.218581 | 0.85941 | -0.443622 | 0.00646031 | 0.056602524 | Non-significant |
| MAPK13 | Mitogen-activated protein kinase 13 | 0.1896425 | 1.14048 | -0.111315 | 0.490599675 | 0.209827936 | Non-significant |
| MAPK9 | Mitogen-activated protein kinase 9 | 0.0490048 | 1.03455 | -0.271971 | 0.369980265 | 0.758953619 | Non-significant |
| MAPKAPK2 | MAP kinase-activated protein kinase 2 | 0.1452071 | 1.10589 | -0.127278 | 0.417692441 | 0.287478648 | Non-significant |
| PTPN6 | Tyrosine-protein phosphatase non-receptor type 6 | 0.0903148 | 1.0646 | -0.106599 | 0.287228733 | 0.359014003 | Non-significant |

Supplementary Table 1: Initial results of the Olink (Proximity Extension Assay) analyzing indicated protein levels in urine of CCM patients compared to controls.
