# Supplementary material for: Lectin-type oxidized LDL receptor-1 as a potential therapeutic target for cerebral cavernous malformations treatment
Source: Front Neurosci. 2024 Aug 21;18:1442110. doi: 10.3389/fnins.2024.1442110 (PMC11371587; doi:10.3389/fnins.2024.1442110)
Supplement: Supplementary file 4 [file Table_2.DOCX]

| **Gene** | **Gene Full name** | **Estimate** | **Fold change** | **conf.low** | **conf.high** | **Adjusted_p value** | **Threshold** |
| --- | --- | --- | --- | --- | --- | --- | --- |
| CD36 | Platelet glycoprotein 4 | 0.6904569 | 1.61379 | 0.0229487 | 1.357965105 | 0.043077406 | Significant |
| LDLR | Low-density lipoprotein receptor | -0.107257 | 0.92835 | -0.615334 | 0.40081969 | 0.669454682 | Non-significant |
| LDLRAP1 | Low density lipoprotein receptor adapter protein 1 | 1.9465749 | 3.85458 | 0.4388058 | 3.454344018 | 0.013133595 | Significant |
| LPA | Apolipoprotein(a) | -1.611454 | 0.32727 | -3.043584 | -0.179323418 | 0.02870968 | Significant |
| LPCAT2 | Lysophosphatidylcholine acyltransferase 2 | 0.9856041 | 1.98014 | -0.11068 | 2.081888734 | 0.07627401 | Non-significant |
| LPL | Lipoprotein lipase | -0.563753 | 0.67654 | -1.04402 | -0.083485804 | 0.022948046 | Significant |
| LRP1 | Prolow-density lipoprotein receptor-related protein 1 | -0.141033 | 0.90687 | -0.486231 | 0.204164457 | 0.410658184 | Non-significant |
| LRP11 | Low-density lipoprotein receptor-related protein 11 | -0.140624 | 0.90713 | -0.529318 | 0.248069313 | 0.465729089 | Non-significant |
| LRP2 | Low-density lipoprotein receptor-related protein 2 | -0.250983 | 0.84032 | -0.864894 | 0.362927799 | 0.41035439 | Non-significant |
| LRP2BP | LRP2-binding protein | -0.027781 | 0.98093 | -0.679539 | 0.623977132 | 0.931209708 | Non-significant |
| LRPAP1 | Alpha-2-macroglobulin receptor-associated protein | 0.5393111 | 1.45328 | 0.0158446 | 1.062777661 | 0.043857846 | Significant |
| LRRC25 | Leucine-rich repeat-containing protein 25 | -0.335082 | 0.79274 | -0.914021 | 0.243857921 | 0.246475775 | Non-significant |
| OLR1 | Oxidized low-density lipoprotein receptor 1 | 1.4576642 | 2.74663 | 0.5224251 | 2.392903253 | 0.003382747 | Significant |

Supplementary Table 2: Initial results of the Olink (Proximity Extension Assay) analyzing indicated protein levels in the plasma of CCM patients compared to controls.
